# Assessment of biochemical biomarkers and environmental stress indicators in some freshwater fish

**DOI:** 10.1007/s10653-024-02226-6

**Published:** 2024-10-03

**Authors:** Salwa M. Abdallah, Reham E. Muhammed, Reda El. Mohamed, Hala El Daous, Dina M. Saleh, Mohamed A. Ghorab, Shaohua Chen, Gharieb S. El-Sayyad

**Affiliations:** 1 Center of Excellence for Toxicological Testing, Department of Mammalian and Aquatic Toxicology, Central Agricultural Pesticides Lab (CAPL), Agricultural Research Center (ARC), Dokki, Egypt; 2 Department of Hygiene and Veterinary Care, Faculty of Veterinary Medicine, Benha University, Toukh, 13736 Egypt; 3 Forensic Medicine and Clinical Toxicology Department, Faculty of Medicine, Assiut University, Assiut, Egypt; 4 Wildlife Toxicology Lab, Dept. of Animal Science, Institute for Integrative Toxicology (IIT), Michigan State University, East Lansing, MI 48824 USA; 5 National Key Laboratory of Green Pesticide, Integrative Microbiology Research Centre, South China Agricultural University, Guangzhou, 510642 China; 6 Medical Laboratory Technology Department, Faculty of Applied Health Sciences Technology, Badr University in Cairo (BUC), Cairo, Egypt; 7 Department of Microbiology and Immunology, Faculty of Pharmacy, Galala University, New Galala City, Suez, Egypt; 8 Department of Microbiology and Immunology, Faculty of Pharmacy, Ahram Canadian University (ACU), Giza, Egypt

**Keywords:** Fish, Biomarkers, Pesticides, Persistent organic pollutants (POPs), Aquatic ecosystem

## Abstract

The mechanism by which an organism can adapt to subtle environmental changes is predicated on modifications to biochemical processes within the cellular metabolism in response to such changes. Changes in these processes have the potential to induce alterations in cellular structures and tissue organization, as well as establish a causal link between fluctuations in these parameters and stressors exposure. This investigation's main goal and innovation is to evaluate the environmental stress indicators in the aquatic ecosystem of Lake Qarun. Pesticide residues in freshwater fish should be the primary focus of evaluation of environmental stressor concentrations, since they serve as bioindicators at different times and places on a spatiotemporal scale. A thorough analysis of suggestive biochemical biomarker reactions should also be conducted. The effects of environmental stressors, specifically pesticide contamination in Qarun Lake, have been observed and investigated in relation to two fish species: *Solea aejabtiaca* and *Oreochronis niloticus*. The results of a hazard assessment conducted at six sampling sites using spatio-temporal data revealed elevated mean values for the pesticides, persistent organic pollutants (POPs), organochlorines, organophosphates, and pyrethroids that were detected. A multi biomarker approach facilitates a more comprehensive understanding of stress responses induced by exposure to pollutants. As a result, the activities of the biochemical biomarkers CYP-450, GST, GSH, and LDH in the blood and liver of fish samples were found to be notably elevated. The suitability of the identified variables for biomonitoring of aquatic pollution was validated, and the data unveiled variations in sensitivity among species, implying that Nile tilapia could potentially function as a bioindicator with high sensitivity. The findings were correlated with the concentrations of detrimental organochlorines, organophosphorus, and pyrethroids in the muscles and gills. The data indicates that pollutants linked to agricultural wastes, runoff, and municipal effluent may be discharged into the lake ecosystem. Consequently, to safeguard the environment, it is essential to enforce and implement policies, acts, and regulations that already exist. Assessing the effects of additional environmental stressors on aquatic ecosystems is another way in which biomarker screening with an integrative approach improves our comprehension of how toxicants impact various levels of biological organization and is particularly useful in realistic environmental exposure scenarios.

**Supplementary Information:**

The online version contains supplementary material available at 10.1007/s10653-024-02226-6.

## Introduction

Aquatic life is most at risk from environmental toxins, including heavy metals and pesticides (Okereafor et al., 2020). In developing nations like Egypt, there is a significant growth in domestic, agricultural, and industrial sectors. Regrettably, pesticides are widely employed without due consideration for the potential threats they pose to the biosphere (Rajak et al., 2023). While agricultural pesticides are designed to operate with a high level of confidence and minimum harm to human health and the environment, the available literature does not consistently support this assertion (Carpy et al., 2000). Aquatic ecosystems are increasingly impacted by agricultural pesticides, which enter water bodies through runoff, leaching, and atmospheric deposition (Blann et al., 2009). These contaminants pose significant risks to non-target species and ecosystem health (Schwarzenbach et al., 2006; Stehle & Schulz, 2015).

Pesticides commonly found in aquatic environments include organochlorines, organophosphates, and pyrethroids. Organochlorines (e.g., DDT, endosulfan) are known for their persistence and bioaccumulation potential (Smith, 2012). Organophosphates (e.g., chlorpyrifos, malathion) are less persistent but highly toxic, affecting the nervous system by inhibiting acetylcholinesterase (Fulton & Key, 2001). Pyrethroids (e.g., cypermethrin, permethrin) are widely used due to their effectiveness and lower mammalian toxicity but are extremely toxic to aquatic organisms (Schleier & Peterson, 2011).They can have harmful effects on numerous creatures besides pests (Bradbury & Coats, 1989; Rajak et al., 2023). According to Fernández (2021**),** out of the 4.19 million metric tons of pesticides consumed worldwide in 2019, the United States accounted for 408 thousand tons, Brazil for 377 thousand tons, and Argentina for 204 thousand tons. China ranked first with 1.76 million metric tons. There has been 20% rise in the use of pesticides in developing nations in the Southeast every year. Among African nations, only Mauritius, the Seychelles, and Egypt used pesticide levels higher than the world average on their crops in 2020 (Shattuck et al., 2023).

Such pollutant stress typically induces a cascade of biological responses in organisms that are exposed; each pollutant may serve as a biomarker for assessing the detrimental responses to pollutants. Chronic stress on non-target species can be investigated by identifying physiological, biochemical, and/or molecular alterations that serve as "early warning" signals for monitoring environmental quality (Hernández-Moreno et al., 2014).

Biomarkers determine the toxicity and effects of heavy metals ingested at a specific level by sample organisms. Studies of biomarkers make it possible to control pollution even at low concentrations. Beginning at the molecular, cellular, and community levels, biomarker research can be conducted (Kadim & Risjani, 2022). Using biochemical biomarkers is essential and may have a strong relationship with the negative species-level effects, and the most precise risk assessment is achieved by combining chemical residue quantification (exposure evaluation) with a biological evaluation (Dhama et al., 2019). Due to the presence of multiple stressors and the complexity of ecosystems, evaluating the effects of contaminants on the health of aquatic organisms and ecosystems is challenging (Hylland et al., 2017). The ability of different pollutants to mutually affect toxicity or even act synergistically can have an impact on the assessment of adverse effects (Vasconcelos et al., 2019).

When exposed to contaminants in aquatic ecosystems, fish usually exhibit toxicological effects first (Mahboob et al., 2015). In terms of ecological significance, fish are regarded as a useful biomarker due to their lower detoxification enzyme activity (e.g., mono-oxygenases) compared to humans, which allows for greater bioaccumulation of toxicants in fish (Monnolo et al., 2023). Critical to routine biomonitoring studies is the selection of sensitive fish species. Notably, Tilapia (*Oreochronis Niloticus*) species are becoming the most common fish species used in biomonitoring surveys in tropical countries. Many publications (Guyon et al., 2012; Hardy & Kaushik, 2021; Lawal, 2021) report that the increasing use of tilapia in environmental health studies and its massive use in aquaculture are generating a substantial volume of basic information regarding genetics, metabolism, and physiology that will aid in the validation of biomarker responses.

Typically, the most sensitive biomarkers in fish fluctuate based on the concentration and activity of biotransformation enzymes. Cytochrome P450 (CYP 450) is essential for the oxidative metabolism and biotransformation of a vast array of endogenous and exogenous compounds. In addition, it is regarded as one of the most crucial phase I biotransformation enzymes (Ibor et al., 2020). The CYP 450 family is extensively reported as an environmental contamination biomarker in aquatic ecosystems (Bhutia et al., 2014), as it is first detectable with a quantifiable response to adverse environmental changes and is highly sensitive to various xenobiotics. In addition, aquatic species possess a diverse array of antioxidant enzymes that comprise antioxidant defenses, such as enzymatic components (e.g., superoxide dismutase (SOD), catalase, glutathione peroxidase (GPx), glutathione S transferase (GST), and small molecule antioxidants (e.g., glutathione; GSH (Hook et al., 2014). Oxidative stress is part of the aging process that occurs in all living cells as reactive oxygen species (ROS) cause cell and tissue damage (Jomova et al., 2023), but exposure to environmental stressors, such as pesticides and metals, increases the rate of ROS in aquatic organisms (Taylor & Maher, 2010). Also, lactate dehydrogenase (LDH) plays a crucial role in the biochemical adaptation of aquatic organisms to chemical stress-induced decreased oxygen levels and increased energy demand. Diamantino et al., (2001), suggested that this enzyme may be a sensitive criterion in both laboratory and biomonitoring investigations.

Lake Qarun is an inland confined basin in an arid region, one of Egypt's five largest lakes, and a popular fishing and tourism destination (Omran & Negm, 2020). The lake is regarded as the primary drainage reservoir for agricultural effluent in Fayoum Province. According to previous reports, the lake receives about 400 million cubic meters of agricultural wastewater drainage annually, which roughly balances the amount of lake water lost annually by evaporation, resulting in a progressive increase in salinity and negative effects on the lake environment, such as its fauna and flora (Dunlop et al., 2005; El-Shabrawy & Dumont, 2009; El-Shabrawy & Gohar, 2008). In addition, the pesticide distribution pattern is one of the lake's main components. Mansour et al., (2004), determined that sediment > fish > water contained the highest concentrations of estimated total pesticide residues. It was determined that pesticide contamination in an ecosystem such as Qarun Lake could be regarded an additional factor endangering this ecosystem; therefore, pesticide residues must be continuously monitored.

In light of the aforementioned, this investigation's main goal and innovation is to evaluate the environmental stress indicators in the aquatic ecosystem of Lake Qarun. Pesticide residues in freshwater fish should be the primary focus of evaluation of environmental stressor concentrations, since they serve as bioindicators at different times and places on a spatiotemporal scale. A thorough analysis of suggestive biochemical biomarker reactions should also be conducted.

## Materials and methods

### Sampling locations

**Fig. 1:**
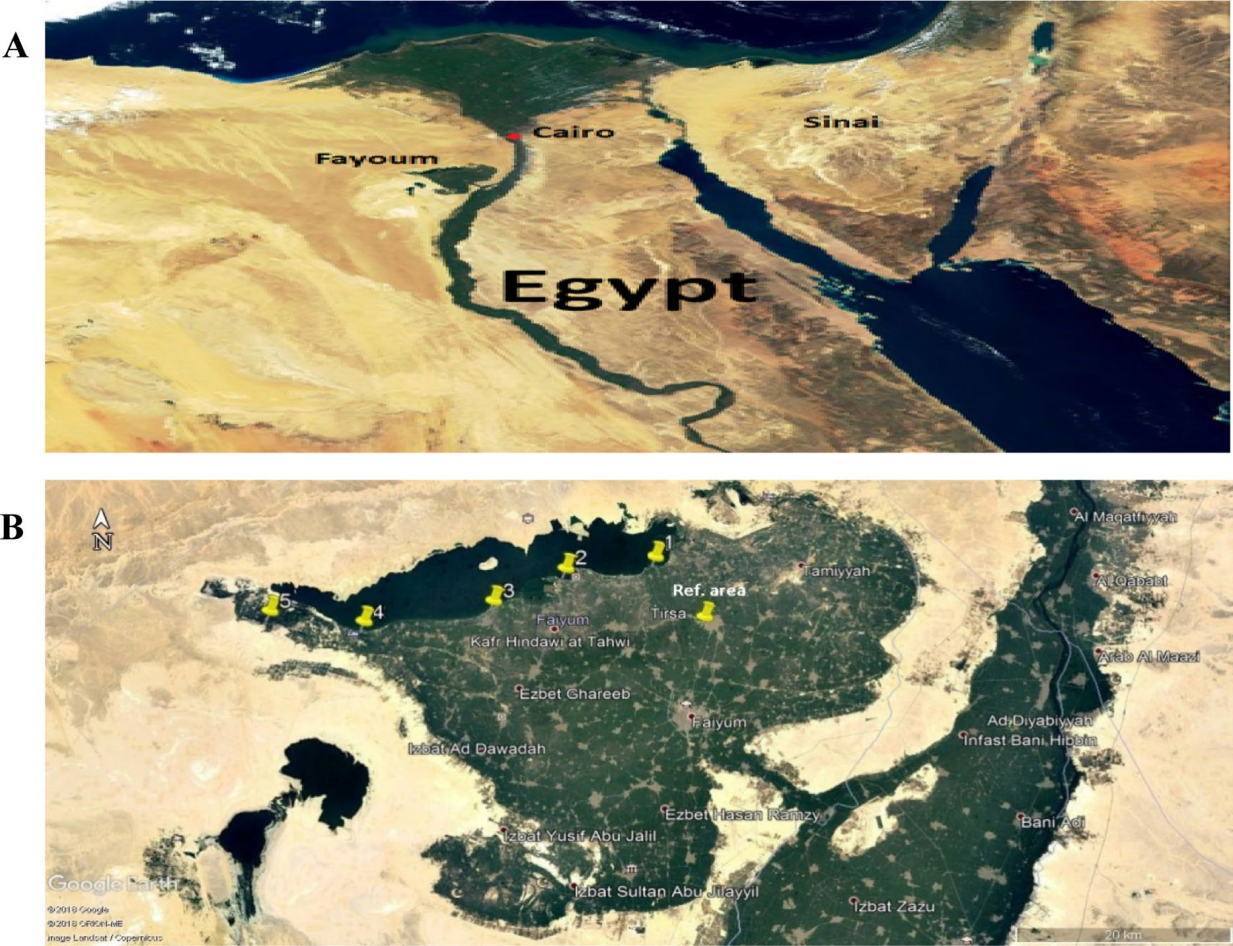
**A** The red circle is indicating location of Qarun Lake in Fayoum Depression; **B** The small five red circles are indicating the sampling locations at Qarun Lake. Fish hatchery at Tersa district taken as a Reference area

Lake Qarun is in the Fayoum Depression on the edge of Egypt’s Western Desert, approximately 80 km southwest of Cairo and has a surface area of 226 km^2^. The lake is 40 km in length, 5.7 km in width, and 43 m below mean sea level. It has an average depth of 4.2 m (Fig. 1A) (Baioumy et al., 2011). The lake receives untreated agricultural and domestic effluent drainage water from Fayoum Province, nearby villages, adjacent cultivated lands, and industrial drainage from Kom-Oshem industries. Most of the drainage water enters the lake either directly or through the two main sewers, El-Batts in the northeastern corner and El-Wadi near the middle of the southern shore (Barakat et al., 2013).

The study included five distinct locations along the lake's southern shore close to residential areas as well as five fish farms close to the selected sites on the lake shore (there are 209 fish farms distributed along the lake shore and fed by the lake's water) compared to a fish hatchery in the Tersa district used as a reference area on the map depicted in (Fig. 1B). With the assistance of local fishermen, tilapia (*Oreochronis Niloticus*) and mousa (*Solea Aegyptiaca*) were collected seasonally (summer & winter) from various locations on Qarun Lake in 2019. *Tilapias* and *Mousa* five samples each were collected from each lake location (total 100 fish samples during summer and winter), whereas only tilapia was available in the adjacent fish farms (total 50 fish samples during summer and winter).

### Sampling procedures

Local fishermen used mesh nets to collect healthy, robust fish samples of two species (*Oreochronis Niloticus* and *Solea Aegyptiaca*) from the designated sampling sites of Qarun Lake. After capture, fish were transferred to enormous vessels containing lake water that had been aerated. According to UNEP 2007, fish sampling was conducted within a few days of capture (Dural et al., 2007). To prevent the stress caused by handling, the fish were mildly anesthetized. Blood was extracted from the caudal vessels using untreated, sterile plastic syringes with 21-gauge needles for biochemical analysis (protein and LDH measurements) (Adham et al., 2001). One hour was permitted for the blood to clot at room temperature. The separation of serum by centrifugation at 14,000 Xg for 5 min. For each sampling location, samples were stored at -20 °C until subsequent analysis. On isolated microsomes, biotransformation assays for the estimation of CYP-450, hepatic GSH content, and GST activity were conducted. All procedures in this study adhered to the protocol for the care of laboratory animals, and the capture of fish was authorized by local authorities.

### Analytical procedures

#### Determination of pesticide residues in fish gills and tissues

Pesticide residues were extracted from samples in accordance with AOAC, 1995-described and analyzed using Gas Liquid Chromatography (GLC). Biochemical tests, such as LDH activity, GST, and GSH levels, were conducted on blood serum and liver microsomes using established protocols. procedures. Fish gills or flesh (50 g) were blended for 2 min with anhydrous sodium sulfate in 150 ml of 40–60 °C petroleum ether (Lehotay, 2007). The extract was re-extracted with petroleum ether twice more. The combined extract was passed over anhydrous sodium sulfate, and the eluent was evaporated in a rotary evaporator until dry, prior to defatting the extract with a 1:1 (v/v) mixture of saturated hexane and acetonitrile. The final extract was loaded onto a florisil column and eluted using solvent systems 6, 15, and 50 weight percent (v/v) diethyl ether in petroleum ether. Gas Liquid Chromatography (GC Hewlett-Packard; BC model 6890) was utilized for the determination of pesticide residues. Under the temperature program (160–210 °C at a rate of 50 °C/min for 10 min, followed by 240 °C at a rate of 50 °C/min for 20 min), PAS-5 and PAS-1701 columns were utilized. The injection and detector temperatures were set to 160 °C.

The flow rates of hydrogen, oxygen, and nitrogen were 75, 100, and 11.7 ml/min, respectively. Identifying pesticide residues by comparing their retention time (RT) to that of authentic standards and confirming the identification with a confirmatory column. Before analyzing samples, blanks were determined, and their values were subtracted from the results. All pesticides had a limit of detection (LOD) of 0.05 μg/kg and a limit of quantitation (LOQ) of 9 μg/kg. The recoveries for OC compounds ranged from 70 to 98%, while those for OP compounds ranged from 78to 89%, additionally, the concentrations of pollutants were calculated. Table 1 showed the Percent recovery of pesticides from fortified fish samples by GC-ECD.

**Table 1:** The percentage of pesticides recovered from fortified fish samples using GC-ECD

Pesticide	*Tilapia fish*	*Mousa fish*	Pesticide	*Tilapia fish*	*Mousa fish*
α-BHC	90.4	94.2	Endosulfan	82.4	80.4
β-BHC	81.6	82.1	p,p-DDE	83.3	80.6
γ-BHC	90.5	95.8	p,p-DDD	98.2	94.7
δ-BHC	92.3	89.7	p,p-DDT	95.2	78.5
Heptachlor	80.1	92.7	Chlorpyrifos	80	78
Heptachlor epoxide	89.2	82.2	Chlorpyrifos-methyl	83	82
Aldrin	80.4	70.5	Dimethoate	88	89
Endrin	90.1	94.8	Malathion	87	88
γ-Chlordane	82.1	79.8	Profenophos	89	89

*NB. (Each value represents the average of 5 sample analysis)*

#### Quality assurance and quality control

Quality assurance measures were implemented throughout the study to ensure data reliability. These measures included calibration of instruments using certified reference materials, running procedural blanks, and conducting replicate analyses. The Limit of Detection (LOD) for pesticide residues was established at 0.05 μg/kg, and the Limit of Quantitation (LOQ) was set at 9 μg/kg. These values will be clearly highlighted in the revised tables for clarity.

#### Biochemical tests on blood serum

##### Protein content

Using a standard commercial kit, total protein was determined colorimetrically based on Biuret reactions employing the recommendations of Weichselbaum (1946).

##### LDH isozymes

Following the procedure of Davis et al. (1973), 50 µl of serum was mixed with an equal volume of glycerol and two drops of bromophenol blue (0.01%) before being separated by electrophoresis on polyacrylamide gels. After loading the samples, the electrode buffer (0.05 M Tris–glycine, pH 8.3) was added gently, and electrophoresis was conducted at 4 °C, 1.5 mA for 15 min, and then at 3 mA. The gels were stained for LDH following the method of Rosalki (1974), using the staining solution (2.5 ml of 1 M Tris-HC1 buffer, pH 8.3, 0.5 ml of 1N lithium lactate, 80 mg of nicotinamide adenine dinucleotide (NAD), 1.2 mg of phenazine methosulphate (PMS), 0.8 mg of p-nitroblue tetrazolium chloride (NBT), and 47 ml of water). The gels were incubated with the staining solution at 37 °C for 15 min, after which the LDH bands took on a violet hue.

#### Tests of liver biotransformation

##### Isolation of microsomes

After the blood was collected, the fish were euthanized. The abdominal cavities were immediately dissected, and the livers were removed, rinsed with a cold solution of 0.1 M potassium phosphate buffer at pH 7.4, dried, weighed, and cooled on ice. All the subsequent procedures were conducted at a temperature of 4°C. A 33% (w/v) crude homogenate was produced by homogenizing 1g of liver with volumes of 0.1 phosphate buffer, pH 7.4, using a Teflon piston and five to eight strokes. To isolate intact cells, nuclei, and mitochondria, the homogenate was subjected to centrifugation at a force of 11,000 Xg for 20 min at 4 °C. To separate microsomal particles, the solution remaining after centrifugation was subjected to further centrifugation at a force of Xg for 60 min at 4 °C. The microsomal granules, which serve as a source of enzymes, were reconstituted in a 0.1 M phosphate buffer with a pH of 7.4 and stored in an ice bath.

##### Assessment of CYP-450

The determination of liver microsomal P-450 was conducted utilizing the methodology described by Omura and Sato in 1964. To reduce the concentration of hepatic microsomal fraction suspended in 0.1M phosphate buffer at pH 7.4, a few crystals of sodium dithionite were added to each cuvette and the mixture was agitated. Subsequently, carbon monoxide was introduced into the sample cuvette at a rate of 20–30 bubbles per min. The resulting spectrum of binding, spanning the wavelength range of 400–500 nm, was obtained by deducting the baseline. By applying an extinction coefficient of 91 cm^−1^ mM^−1^, it was possible to ascertain the concentration of hemoprotein complex. As described by Lowry et al. (1951), the protein content of the microsomal fraction was ascertained utilizing bovine serum albumin as a standard.

## Levels of GST and GSH

The spectrophotometric activity of GST was determined at 340 nm using the method described by Habig et al. (1974). For measuring reduced glutathione concentrations, Ferrari et al. (2007), modified the method developed by Sedlak and Lindsay in 1968. By measuring the absorbance of reduced chromogen at 412 nm, the concentration of GSH could be determined.

### Statistical analysis

Using SPSS, descriptive statistical analysis was conducted. The data was displayed as means ± standard error. According to Snedecor and Cochran (1969) (D'AGOSTINO RB., 1971), an analysis of variance (one-way ANOVA) was performed, followed by Dunnett's test. Two significance probabilities were reported: *P value < *0.05*; **P value < *0.01*.

## Results

Due to the pervasiveness of pollution, both the physical systems and living organisms that inhabit our ecosystem have experienced instability, disorder, injury, or distress. For an accurate assessment of environmental risk, it is optimal to combine the quantification of chemical residues (exposure assessment) with a biological assessment, which evaluates ecosystem effects directly on the exposed organism. The percentage of pesticides extracted from fortified fish samples via GC-ECD is presented in Table 1.

### Pesticide residue analysis

Table S1 presents the temporal and spatial distribution of organochlorine concentrations in fish samples, with higher concentrations observed during the summer. Endosulfan concentrations ranged from 19 to 1338 ng/g, consistent with the values provided in Table 2. All discrepancies in units between the text and tables have been resolved, ensuring consistency in using ng/g for all concentration measurements.

**Table 2:** Means of organochlorines and pyrethroids (ng/g) in fish samples; *O. Niloticus, from Fayioum governorate 2019,* (N = 100 for each season)

	Summer				Winter			
Pesticide	Qarun lake		Fish farms		Qarun lake		Fish farms	
	Gills	Muscles	Gills	Muscles	Gills	Muscles	Gills	Muscles
α-BHC	^*^ND				ND			
β-BHC	ND			0.56 (0.38– 1.75)	ND			
γ-BHC	ND	1.03 (0.5–2.11)	ND		ND			
δ-BHC	ND	0.45 (0.1–1.26)	6.00 (2.11–14.30)	1.50 (0.46–3.96)	ND	16.50 (2.14–40.19)	ND	13.95 (6.52–41.20)
Aldrin	ND				ND			
Endrin	ND	4.66 (2.15–6.46)	12.65 (11.39–19.18)	5.02 (3.23–8.61)	ND			10.76 (4.61- 40.81)
Dilderin	ND				ND			
Heptachlor	1.80 (0.90–3.12)	4.39 (0.30–15)	3.40 (2.40–6)	1.75 (0.75–8.20)	ND			
Hept. epoxide	ND			3.89 (0.35–9.84)	ND			
p,ṕ -DDE	ND			0.10 (0.02–6.91)	ND	3.17 (0.58–9.16)	ND	
p,ṕ -DDD	ND	0.83 (0.23–1.51)	6.37 (2.11–31.15)	0.49 (0.23–16.75)	ND			
p,ṕ -DDT	ND				139.08 (60.13–210.91)	ND		36 (15.42–61.30)
Endosulfan	9 (6–17)	10 (1.5–18)	20 (12–130)	3.65 (0.95–16)	105 (26–464)	ND		
Chlordan	ND	0.825 (0.15–2.50)	ND	6.00 (0.50–40.50)	ND	21 (4.91–80.11)	ND	19.50 (7.50–40.50)
Sumicidin	ND	102.75 (91.5–714)	ND					
Meothrin	ND				ND			
Cypermethrin	ND			157 (103–761)	96 (24–218)	ND		
Permethrin	1383 (1272–1694)	ND	3012 (1182–9842)	442.5 (218–720)	ND	347 (189–619)	2036 (1120–5480)	ND
Fenvalerate	ND				37.8 (19.72–81.90)	237 (123–890)	ND	
Deltamethrin	ND				6436 (3821–11,108)	ND		

*ND: Not Detected, Mean concentration (Min–Max)

Table 2 illustrates the temporal and spatial distribution of organochlorine concentrations (OCs) in fish specimens of *Solea Aegyptiaca* and *Oreochronis Niloticus* that were collected from Qarun Lake. For both fish species, summer concentrations of OC residues were greater than winter concentrations.

**Fig.2:**
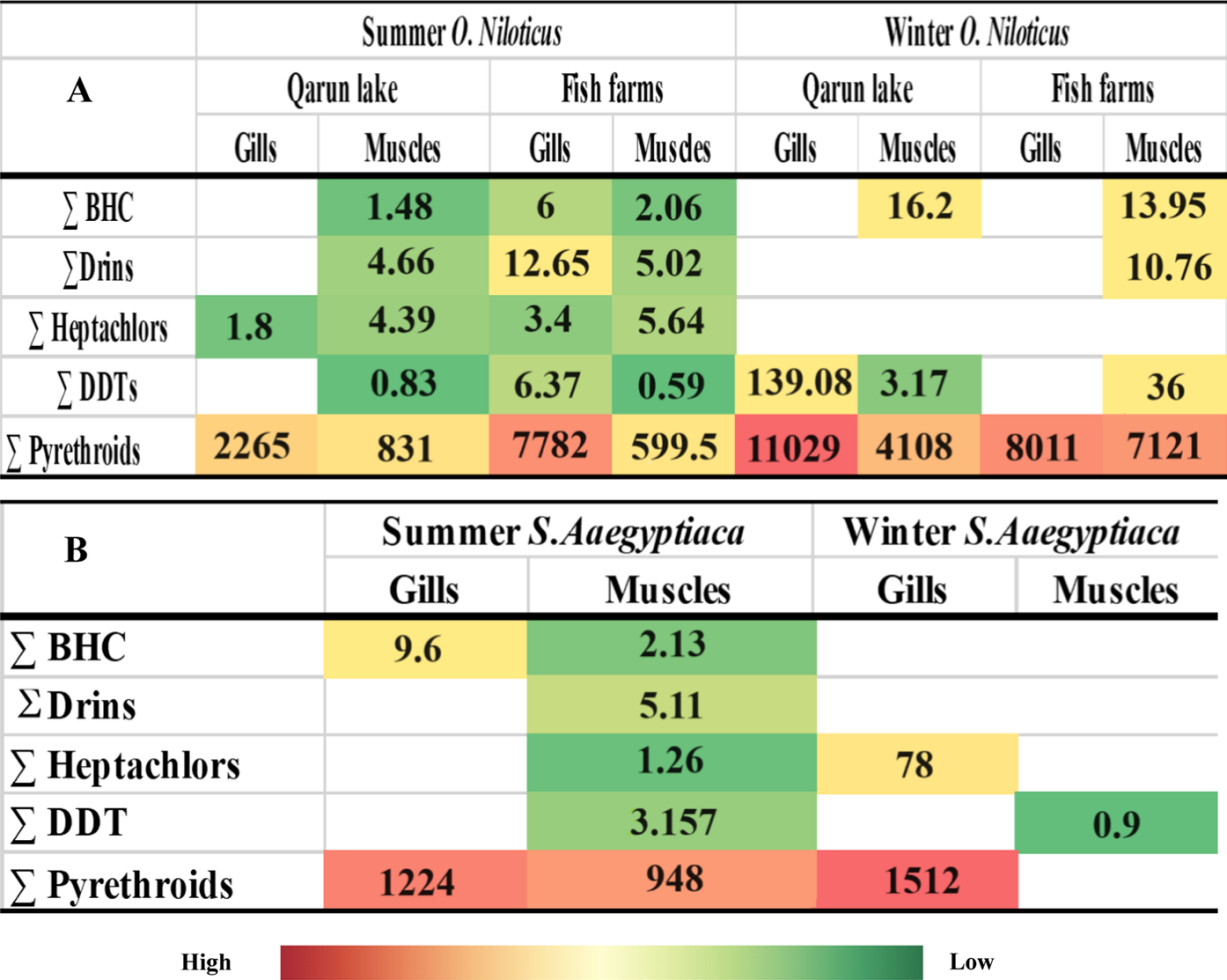
**A**: *Heat map of sum means of organochlorines and pyrethroids (ng/g) in fish samples; O. Niloticus, from Fayioum governorate 2019.* **B** Sum Means of organochlorines (ng/g) and pyrethroids (ng/g) in fish samples; *S. egyptiaca* from Qarun Lake during 2019. White colored cells indicated Not Detected, colored cells indicate detected levels

Furthermore, endosulfan exhibited the highest concentration among the identified organic compounds (19–1,338 ng/g), followed by chlordane (0.83–21 ng/g), DDT isomers (0.83–142.25 ng/g), and total BHC concentration (0.45–16.5 ng/g) in *O. Niloticus* samples obtained from Qarun Lake. ∑DDT and endosulfan were significantly present in the gills of *O. Niloticus* during the winter. Both chlordane and ∑BHC were identified in muscle tissue at decreased concentrations during the same season. During the summer, endosulfan was abundant in the musculature and gills of both tested fish species. *S. Aegyptiaca* appeared to contain more POP residues in the summer than in the winter (Fig. 2 A, B).

### Biomarker responses

Significant increases in biomarker activities, including CYP-450, GST, GSH, and LDH, were observed in fish samples from polluted sites compared to the reference site, indicating oxidative stress responses (Table S2). These biomarkers demonstrate the utility of using fish as indicators of environmental health. In both fish species, pyrethroid insecticides were detected more frequently than other types of pesticides (Table 3). *O. Niloticus* detected pyrethroids more frequently in the winter than in the summer, and vice versa for *S. Aegyptiaca*. Summer and winter samples of the same species collected from Qarun Lake contained a lower concentration of OCs and pyrethroids than samples collected from the fish farm closest to Qarun Lake. The levels of total DDT and pyrethroids were higher in winter for lake and farm samples than in summer samples. The gills of *O. Niloticus* were found to be more contaminated with OCs residues than the muscle tissue during the summer. Muscles were found to be more contaminated than gills for most surveyed pesticides, except for total pyrethroids, which had a higher concentration in gills.

**Table 3:** Means of organochlorines (ng/g) and pyrethroids (ng/g) in fish samples; *S.Aaegyptiaca* from Qarun Lake during 2019 (N = 50 for each season)

	Summer		Winter	
	Gills	Muscles	Gills	Muscles
β-BHC	2.40 (1.60–4.91)	0.63 (0.38–0.75)	^*^ND	
δ-BHC	7.20 (2.40–22)	1.50 (0.37–4.72)	ND	
Endrin	ND	5.11 (2.15–19.53)	ND	
Dilderin	ND	ND	ND	
Heptachlor	ND	1.26 (0.30–12.25)	78 (31.55–120.70)	ND
Hepta.epoxide	ND	ND	ND	
p,ṕ -DDE	ND	0.15 (0.11–0.42)	ND	0.90 (0.45–3.27)
p,ṕ -DDD	ND	3.37 (0.30–9.05)	ND	
p,ṕ -DDT	ND	ND	ND	
Endosulfan	9*.*00 (3.00–22.00)	13.80 (1.50–34)	1338 (908.70–1870.50)	ND
Chlordan	ND	5.25 (4.50–26)	ND	
Sumicidin	ND	120.75 (114–177.50)	ND	
Cyhalothrin	ND	502.50 (321.71–840.50)	ND	
Cypermethrin	ND	312 (178.51–763.50)	ND	
Permethrin	1224 (980–2150)	ND	1512 (1105.42–2105.60)	ND
Meothrin	ND	133.50 (98.50–310.80)	ND	

*ND: Not Detected, Mean concentration (Min–Max)

### Seasonal variations in pesticide detection

Seasonal differences in pesticide levels were noted, with higher concentrations detected during summer due to increased agricultural runoff. Table 4 includes data on mean concentrations, concentration ranges, and detection frequencies for all monitored pesticides.

**Table 4:** Mean levels of organophosphorus pesticides (ng/g) in fish samples from Fayoum Governorate during 2019 (N = 150 for each season)

Pesticide	Tilapia Fish (*O. niloticus*)								Mousa Fish (*S. aegyptiaca*)			
	Summer				Winter				Summer		Winter	
	Mean (Min–Max)				Mean (Min- Max)				Mean (Min–Max)		Mean (Min–Max)	
	Qarun Lake		Fish farm		Qarun Lake		Fish farm		Qarun Lake		Qarun Lake	
	Gills	Muscles	Gills	Muscles	Gills	Muscles	Gills	Muscles	Gills	Muscles	Gills	Muscles
Chlorpyrifos	^*^ND				28.40 (12.6—41.50)	62.73 (27.85–109.5)	ND		ND			
Chlorpyrifos-methyl	ND				72.00 (46.7- 120.45)	ND	ND		ND			
Dimethoate	ND				93 (24—462)	4.50 (1.76–16.95)	ND		ND			
Malathion	ND				11.63 (1.46—51.80)	16.65 (10.45–31.75)	179.72 (110.65–317.5)	49.96 (19.4–87.15)	29.15 (7.46–51.7)	14.69 (2.75–45.6)	165.16 (105.9–215.65)	44.08 (15.6–76.5)
Profenophos	ND				105.00 (81.6–198.54)	8.25 (2.87–24.19)	21 (9.35—42.72)	6.00 (2.10–18.45)	ND	ND	132.00 (109.5–185.3)	6.75 (1.76–15.42)

*ND: Not Detected, Mean concentration (Min–Max)

OPs were detected more frequently in winter samples of *O. Niloticus* than in summer samples, but the concentration of contaminants was higher in winter in Qarun Lake than in the fish farm. Samples from Qarun Lake were more contaminated than those from fish farms, especially with chlorpyrifos, chlorpyrifos-methyl, dimethoate, malathion, and profenophos for both fish species, with gills typically being more contaminated than muscle tissue. According to Tables 4, and S3, malathion concentration in Tilapia is higher in samples from fish farm compared to those from Qarun Lake.

In the current investigation, LDH isozyme activities were elevated in the blood serum of *O. Niloticus* at all examined sites. The highest isozyme activity values were recorded, specifically at Area (1), for all LDH patterns. Serum total protein levels were significantly lower in fish from Area (3) compared to other sites.

As it relates to biotransformation enzymes: CYT P450: Total CYP 450 was substantially induced in all collected samples, with higher induction observed in areas 1, 2, and 5 compared to reference area samples (Table 5). The data presented here demonstrate the effect of pesticide residues on oxidative stress biomarkers (reduced glutathione, GSH, and glutathione-S-transferase, GST) in *O. Niloticus* collected from Qarun Lake. As a phase II class of enzymes that are the predominant cytosolic defense systems responsible for protecting cellular components from a variety of toxic effects and oxidative stress, GST's activity was found to be significantly elevated at all sites examined. In comparison to the reference area, area (1) exhibited the maximum level of activity (120%), and area (3) exhibited the lowest level of activity (5%). A rise in GST activity was concurrent with a decline in GSH, which was more pronounced in samples collected from the area (1). In addition, GSH showed a lesser decrease in area (2). GSH serves a central role in the antioxidant system, and its depletion is regarded as an important biomarker of pollutant-induced oxidative stress in fish.

**Table 5:** Biomarker responses in freshwater fish; *O. niloticus* from Qarun lake, Fayoum Governorate (N = 55)

Locality	Biomarker response (Mean ± S.E)								
	Cytochrome-P450 (nmoles/mg protein)	GSH content (unit/mg protein)	GST activity (unit/mg protein)	% LDH isozymes fraction					Protein content (g/dl)
				LD1	LD2	LD3	LD4	LD5	
Area 1	10.11 ± 0.59***	4.12 ± 0.30***	2.54 ± 0.021***	58.6 ± 8.90	53.90 ± 10.80	33.00 ± 1.09	23.80 ± 5.02	21.70 ± 3.29	6.80 ± 0.18
Area 2	9.52 ± 0.56***	5.48 ± 0.72*	2.27 ± 0.097***	46.1 ± 15.60	50.2 ± 9.00	27.70 ± 4.85	23.90 ± 2.56	15.90 ± 6.00	7.04 ± 0.35
Area 3	4.24 ± 0.53***	4.93 ± 0.74*	2.01 ± 0.147***	36.26 ± 12.50	39.40 ± 5.80	24.60 ± 2.36	22.00 ± 6.30	15.20 ± 3.67	6.38 ± 0.35
Area 4	2.53 ± 0.72**	4.59 ± 0.45***	2.17 ± 0.092***	33.90 ± 6.60	32.20 ± 3.10	24.00 ± 5.66	15.30 ± 3.90	12.30 ± 3.93	6.78 ± 0.19
Area 5	7.21 ± 0.39***	4.00 ± 0.58***	2.02 ± 0.088 ***	33.5 ± 3.80	36.50 ± 13.40	23.30 ± 3.67	14.10 ± 0.32	11.70 ± 1.45	6.76 ± 0.31
Ref. Area	1.07 ± 0.19	7.80 ± 0.62	1.17 ± 0.11	31.00 ± 3.50	34.90 ± 2.19	21.40 ± 1.38	10.80 ± 1.27	7.90 ± 0.78	7.12 ± 0.19

Data represent 5 locations and 5 fish samples per each locality and detected response represents mean of biomarker concentration in summer and winter

*Significant difference at *p* < *0.05*

**Significant difference at *p* < *0.01*

***Significant difference at *p* < *0.001*

## Discussion

The study identified seasonal variations in pesticide levels, likely influenced by agricultural practices and climatic conditions. The higher pesticide concentrations detected in summer can be attributed to increased runoff following agricultural activities. Differences in pesticide distribution between *O. niloticus* and *S. aegyptiaca*, particularly between gill and muscle tissue, highlight species-specific metabolic and physiological responses to contaminants. The aquatic ecosystem of the lake may be exposed to pesticides through two primary routes: direct infiltration through the application, or indirect ingress via the discharge of effluent and/or agricultural runoff (Belhassan, 2021). Additional contamination could potentially result from factors such as drift, equipment cleansing, or precipitation. Indirectly, the pesticides that have been detected may disrupt the food chain of fish and alter the lake's habitat (Riaz et al., 2021). Presently, OPs, carbamates, synthetic pyrethroids, neonicotinoids, and carbamates comprise most insecticides used in Egypt (Gad Alla et al., 2013). These substances are notoriously toxic but rapidly biodegradable. DDT, lindane, heptachlor, endrin, dieldrin, and aldrin have been identified in numerous environmental compartments, such as sediment, water, and fish, in addition to the Nile River's contribution of numerous OC compounds from African nations (Barakat, 2004), even though the majority of OCs are prohibited in Egypt. During the current study, concentrations of OCs were greater in *S. aegyptiaca* than in *O. niloticus* in the summer and winter, respectively. Additionally, there was an increase in the use of pyrethroid insecticides in both tested fish species, with a particular emphasis on the winter season. Notably, the lake is an ultimate destination downstream that has been contaminated by the massive drainage of agricultural waste from the most populous and developed regions of Egypt via vast rivers.

The capacity of fish to capture and bioaccumulate pollutants at higher concentrations is facilitated by the water's relatively low depth and sluggish flow (Abdallah & Elmagd Morsy, 2013; Kornis et al., 2012). Virtually all pesticides have other ingredients other than the active one, which has the exterminating action and differences in the test-organisms’ responses to the commercial formula, can be attributed to the toxicity of different compounds and surfactants in the commercial formula (Ruiz-Suárez et al., 2015). The study identified seasonal variations in pesticide levels, likely due to different agricultural practices and environmental conditions. The higher concentrations detected in summer correspond with increased agricultural runoff. Differences between *O. niloticus* and *S. aegyptiaca* in gill and muscle pesticide concentrations can be attributed to species-specific metabolic and physiological responses.

Regarding *S. aegyptiaca* samples, the levels of organic compound residues were greater during the summer season compared to winter. Additionally, the muscles of the samples were generally more polluted than the gills, except for total pyrethroids where the gills had a higher concentration. Overall, the *S. aegyptiaca* fish exhibited higher levels of contamination (excluding pyrethroids) compared to the *O. niloticus* fish, specifically with regards to various OCs residues. Sparling et al., (2016) and Tzanetou and Karasali (2022), found that the oil content of fish species regulates the levels of chlorinated hydrocarbon pesticides, such as DDT, dielrin, and endrin. Additionally, the duration required to achieve a sufficient harvest is crucial (Vives et al., 2005), and in the case of *O. niloticus*, it is very short, approximately 6 months. This could be regarded as an additional factor contributing to the accumulation of higher concentrations of OCs in the aforementioned fish.

The lake is contaminated with untreated agricultural and domestic sewage runoff from Fayoum Province, nearby settlements, and surrounding cultivated areas. It is important to note that *O. niloticus* obtained from the fish farms nearest to Qarun Lake showed elevated levels of organochlorines (OCs) and pyrethroids, especially in the gills. The fish farms in the surveyed region are situated in close proximity to cultivated fields and directly collect water from the agricultural drainage originating from these fields. This may elucidate the contamination of farmed fish, particularly with organophosphates (OPs).

The results of our study align with previous research conducted on Qarun Lake, which showed that the levels of contamination from various organic compounds (such as DDT, its isomers, BHC, lindane, heptachlor, aldrin, dieldrin, and endrin) were generally higher during the summer compared to the winter. Additionally, the levels of OPs insecticides (such as malathion and dimethoate) were also found to be higher during the summer (Abou-Arab et al., 1995).

In addition, it was reported that the obsolete organochlorine compounds (such as HCB, lindane, heptachlor, heptachlor epoxide, aldrin, dieldrin, endrin, and DDT analogues), along with some of the currently used organophosphates (such as malathion, dimethoate, pirimiphos-methyl, profenofos, and diazinon), were found in a majority of the water and fish (specifically *Mufil sp*. and *Tilapia sp.*) samples collected from Qarun Lake. The distribution pattern of pesticides in the main components of the lake was as follows: sediment > fish > water. Additionally, fish samples collected from the Turkish aquatic ecosystem were found to contain the degraded product of dichlorodiphenyldichloroethylene (pp-DDE), endosulfan, and heptachlor, as reported by Topal and Onac in 2020.

Pesticide-induced water pollution can have negative impacts on aquatic ecosystems. These pollutants can accumulate in organisms through gills and the food chain, leading to increased fish mortality and reduced fish production. Ultimately, humans can be exposed to these pollutants through consuming contaminated fish, which can result in reproductive and growth abnormalities, as well as adverse effects on human and animal health, such as cancer, mutagenic effects, neurological disorders, and immunological problems (Al-Ghanim, 2014; Arrebola et al., 2012; Dang et al., 2016; Mahboob et al., 2015; Wang et al., 2015).

The presence of stress factors and inappropriate environmental conditions can be indicated by changes in fish blood biochemistry (Abdel-Tawwab et al., 2019; Barcellos et al., 2004; Fırat & Kargın, 2010). As a biomarker for tissue injury in fish, blood biochemical variables, specifically LDH and its isozymes and protein content, were examined in the current investigation (Gabriel et al., 2012; Nathan et al., 2006). The higher rate of lactate conversion to pyruvate and subsequently to glucose may be the cause of the elevated LDH activity seen here (Hori et al., 2006). Importantly, LDH controls the final stage of the anaerobic glycolytic pathway, which is responsible for the metabolism of carbohydrates. Increased levels of LDH signal an adaptive reaction to its leaking into the blood stream owing to the presence of water toxicity, and any shift in the level of LDH activity reflects on to the metabolic alterations in the affected tissues (Fetoui et al., 2009). The current study's detection of LDH's varying response to environmental stressors suggests the application of this biomarker in aquatic ecosystem health surveillance. Our data demonstrated that exposure to environmental stresses, especially at areas 1 and 2, had changed all LDH isozymes in all fish samples that were tested. Comparing all isozymes to samples taken from the reference location, they all displayed good sensitivity.

Following pesticide exposure, the same pattern was previously reported in the blood of African catfish and Nile tilapia (Adedeji et al., 2009). Additionally, the Nile Rosetta and Damietta branches have recorded an environmental pollution with pesticides and other sources (Osman & Kloas, 2010), which may contribute to the rise of LDH and other enzyme concentrations in fish blood. According to reports, increases in the activity of serum enzymes are a direct indicator of significant pathologic changes or injury to the liver (Bhattacharjee et al., 2019). The unusual rise in serum enzyme levels was previously explained by Osman (2012) (Osman et al., 1758), who observed severe cellular deterioration and necrosis in the livers of Nile tilapia taken from the downstream Nile River.

Since serum proteins have several roles that are crucial for the control of water balance, an initial electrolyte imbalance is a common manifestation of stressors (Wedemeyer & Yasutake, 1977). Data showed a drop in total protein, which indicated liver dysfunction brought on by a higher pesticide-related aquatic burden. Low protein level affects colloid osmotic pressure and is a sign of disturbed water balance, injury to the kidneys and liver, and hemodilution (Larsson et al., 1985). In order to assess the reaction to stressors and, as a result, the rising demand for energy, the impact of toxicants on fish's total protein content has been taken into account (Hadi et al., 2009).

As evidence of biotransformation and induction of cytochrome P-450, total CYP 450 was significantly induced in all collected samples with higher induction seen in areas 1, 2 and 5 when compared to samples collected from reference area (Table 5). Most lipophilic chemicals, including pesticides and environmental pollutants and naturally occurring compounds undergo enzyme-mediated oxidative, hydrolytic, or conjugative biotransformation in liver and in extra hepatic tissues, yielding more polar metabolites as a response to toxic chemicals (Akdogan and Sen, 2010). It is likely that some P450s differ in their functions among fish and follow different mechanisms of induction. In consequence, whenever P450 enzymes are used as biomarkers in monitoring programs, one should be cautious with metabolic differences, even among fish species (Bhutia et al., 2015), and the current study clearly demonstrated the usefulness of CYP 450 as an important biomarker for monitoring the aquatic pollution. Previous studies coincide with these findings where CYP1A was the most sensitive isoform towards cyperrmethrin toxicity and CYP3A4 was induced in *Labeo rohita* due to exposure to pyrethroid insecticides in aquatic ecosystems (Rai et al., 2019), and in channel catfish (Stuchal et al., 2006), after exposure to chlorpyriphos and methoxychlor.

Furthermore, one of the most readily activated pathways is the antioxidant defense system where antioxidant response is considered important biomarker for aquatic ecosystem contamination (Alam et al., 2017; Li et al., 2011). Protective antioxidant enzymes like GST, that can detoxify a variety of environmental chemicals, and their non-enzymatic cofactors (e.g. GSH) can be overwhelmed during high levels of tissue oxidative stress, which can lead to the accumulation of metabolic by-products reflecting oxidative damage*.*

The information shown in Table 5 demonstrated that the activity of GST, a phase II class of conjugation enzymes and the main cytosolic defense system that shields cellular components from oxidative stress and other harmful effects, was markedly elevated at every location of investigation. When compared to the reference site, site (1) had the most activity (120%) while site (3) had the lowest activity.

Hepatic GST in *O. niloticus* drew a positive correlation with the elevated loads of heavy metals of all test locations. Elevated activities of GST indicate the ability of fish to adapt to different pollutants as well as defense against oxidative damage and peroxidative DNA products and could point out that cytotoxicity has not yet completely occurred. The present results agreed with other studies that indicated 2–3 folds increase in GST activity (Anderson & Gronwald, 1991; Gadagbui et al., 1996; Johnson et al., 1992), in both gills and digestive gland after exposure to heavy metals. Our data also agree with results of Goldberg et al., 1986 (Goldberg et al., 1986), that indicated active GST system in mussels and levels were responsive to environmental stimuli.

Increase in GST activity was concomitant with a significant decline in GSH which exhibited a higher decline in samples collected from site (1) and a lower trend in site (2). GSH plays a central role in antioxidant system and its depletion is considered as an important biomarker of oxidative stress and is often associated with cytotoxicity in fish caused by pollution via heavy metals and pesticides (Pena-Llopis et al., 2001). The response of this enzyme to the detected environmental stressors may be due to that the concentrations of pollutants reached the threshold level for this protein. A positive correlation was found between pesticide concentrations and alterations in biochemical biomarkers, supporting the use of these biomarkers as indicators of environmental stress. These findings highlight the potential for utilizing multi-biomarker approaches to assess aquatic ecosystem health effectively.

Comparable studies showed that GSH response in fish varies in relation to metal exposure concentration and time, fish age and tissue type, as well as the conflicting field and laboratory toxicity tests in gills and in whole fish for *M. cephalus* following Pb exposures (Kroon et al., 2017) and Cd exposure (Gabriel et al., 2020). Additionally, this biomarker does not necessarily reflect contamination levels after a prolonged exposure time since change in GSH/GSSG is biphasic, with increasing activity occurring up until a certain concentration after which levels decline. The return to normal GSH/GSSG levels may be due to compensation by other enzymes such as superoxide dismutase. Concomitantly, response of GST activity to elevated metals levels in water or sediment was varied due to species-specific responses (Das et al., 2017), tissue-specific responses (Das et al., 2017; Mieiro et al., 2014), different feeding habits (Duarte et al., 2017), seasonal and annual variations (Das et al., 2017), and responses to contaminants other than metals (e.g. organics (Duarte et al., 2017; Fonseca et al., 2011)).

Successful implementation of biomarkers in environmental monitoring systems therefore requires a good understanding of the mechanisms behind these responses where many variables related to pollution and non-pollution may have an additional effect on these enzyme systems and may therefore interfere with biomarker responses. Fish in the environment are many times exposed to complex contaminant mixtures, and chemical pollutants often interact with each other, leading to different biomarker responses than fish exposed to only one contaminant at a time, in what has been called “the cocktail effect” (Celander, 2011; Oost et al., 2003).

Fish display specific modifications as they reach their reproductive stages, such as increases in body fat stores, including hepatic alterations, differences in eating, and changes in sexual hormones (Hauser-Davis et al., 2010; Oost et al., 2003**),** which may in turn influence biomarker responses due to extensive physiological changes that occur in fish during this period (Parente & Hauser-Davis, 2013). Some organism modifications may also occur due to seasonality, since certain fish species change their feeding habits during different seasons, due to the availability of certain food items (Angermeier, 1982). Also, certain contaminants may be more bioavailable during the wet season (i.e., chlorinated contaminants such as PCBs and PBDEs, and metals), since increased water column and sediment dislocations are more frequent during this season, resuspending contaminants adsorbed by the sediment, making them more bioavailable to the biota (Parente & Hauser-Davis, 2013).

From ecological significance point of view; using a multi-biomarker approach could facilitate the elucidation of possible cellular or physiological mechanisms of toxicity due to exposure to several environmental stressors (Liu et al., 2014). Bioindicators that are highly affected by pollutants exposure would be easier for predators to capture, which can facilitate the transfer of the contaminants to higher trophic levels (Maznikova et al., 2024). Therefore, using biomarkers is very useful under environmental realistic exposures and screening multiple biomarkers with an integrative approach, helps to improve our understanding of how toxicants affect different levels of biological organization (Moore et al., 2004), and could be also used to assess the effects of other environmental stressors on aquatic ecosystems (Hook et al., 2014; Lomartire et al., 2021).

## Conclusion

The biomarkers examined in *O. niloticus* in response to the identified environmental stressors were specific and could be utilized as monitoring instruments in environmental protection programs, according to the current findings presented herein. The utilization of a biomarker approach that integrates chemical residues in biological tissues with multiple stress responses implies that the distribution of stressor exposure in Qarun Lake is spatiotemporal. This distribution could potentially explain the toxicity, adverse impacts on fish health, and human well-being. Furthermore, changes in the activities of CYP-450, GST, GSH, and LDH that were detected in fish muscles, along with OCs and OPs in the samples, indicate that these pollutants may have been discharged into the water stream via agricultural runoff and industrial debris into the lake. Assuring an acceptable level of environmental quality and implementing and enforcing policies, acts, and regulations that regulate the discharge of runoffs and sewage into rivers and lakes are essential for mitigating the effects of pollution and promoting environmental protection. The study demonstrates the effectiveness of biochemical biomarkers in detecting environmental stressors in Lake Qarun. Future studies should focus on long-term monitoring, including more diverse biomarkers and additional environmental stressors. Implementing and enforcing regulations to control agricultural runoff is essential for protecting aquatic ecosystems. Future research should aim to expand biomonitoring efforts across different geographical locations and seasons, incorporate advanced analytical methods for detecting emerging contaminants, and explore the application of these findings in policy-making and environmental management strategies.

## Supplementary Information

Below is the link to the electronic supplementary material.

Supplementary file1 (DOCX 29 KB)

## Data Availability

No datasets were generated or analysed during the current study.
