# Supplementary material for: Assessment of biochemical biomarkers and environmental stress indicators in some freshwater fish
Source: Environ Geochem Health. 2024 Oct 3;46(11):464. doi: 10.1007/s10653-024-02226-6 (PMC11449979; doi:10.1007/s10653-024-02226-6)
Supplement: Supplementary file 1 — Supplementary file1 (DOCX 29 KB) [file 10653_2024_2226_MOESM1_ESM.docx]

## Table S1: Pesticide Residue Distribution in Fish Samples

| Pesticide | Mean Concentration (ng/g) | Concentration Range (ng/g) | Detection Frequency (%) |
| --- | --- | --- | --- |
| α-BHC | 0 | ND | 0 |
| β-BHC | 0.56 | 0.38 - 1.75 | 40 |
| γ-BHC | 1.03 | 0.5 - 2.11 | 60 |
| δ-BHC | 0.45 | 0.1 - 1.26 | 25 |
| Endrin | 4.66 | 2.15 - 6.46 | 50 |
| Heptachlor | 1.80 | 0.90 - 3.12 | 30 |
| Heptachlor epoxide | 0 | ND | 0 |
| p'p -DDE | 0.10 | 0.02 - 6.91 | 20 |
| p'p -DDD | 0.83 | 0.23 - 1.51 | 35 |
| p'p -DDT | 139.08 | 60.13 - 210.91 | 80 |
| Endosulfan | 19 | 6 - 17 | 100 |
| Chlordane | 0.825 | 0.15 - 2.50 | 45 |

## Table S2: Biomarker Responses in Fish Samples

| Biomarker | Mean Value (nmoles/mg protein) | Concentration Range | Detection Frequency (%) |
| --- | --- | --- | --- |
| CYP-450 | 10.11 | 7.21 - 10.11 | 100 |
| GST | 2.54 | 2.02 - 2.54 | 100 |
| GSH | 4.12 | 4.00 - 5.48 | 100 |
| LDH | 58.6 | 33.5 - 58.6 | 100 |

## Table S3: Seasonal Variations in Pesticide Concentrations

| Pesticide | Mean Concentration (ng/g) Summer | Mean Concentration (ng/g) Winter | Concentration Range (ng/g) Summer | Concentration Range (ng/g) Winter | Detection Frequency (%) Summer | Detection Frequency (%) Winter |
| --- | --- | --- | --- | --- | --- | --- |
| Malathion | 11.63 | 16.65 | 1.46 - 51.80 | 10.45 - 31.75 | 40 | 60 |
| Chlorpyrifos | 0 | 62.73 | ND | 27.85 - 109.5 | 0 | 80 |
| Dimethoate | 93 | 4.5 | 24 - 462 | 1.76 - 16.95 | 100 | 20 |
| Profenophos | 105 | 8.25 | 81.6 - 198.54 | 2.87 - 24.19 | 60 | 45 |

### 
